# Kinetic model of partial agonism reveals cellular basis of ligand efficacy

**DOI:** 10.1016/j.jbc.2026.113180

**Published:** 2026-05-21

**Authors:** Michael Ritt, Nishaben Patel, Edgardo Sánchez Rivas, Sivaraj Sivaramakrishnan

**Affiliations:** 1 Department of Genetics, Cell Biology and Development, University of Minnesota, Minneapolis, Minnesota, USA; 2 Biochemistry, Molecular Biology and Biophysics Graduate Program, University of Minnesota, Minneapolis, Minnesota, USA

**Keywords:** G protein, G protein-coupled receptor (GPCR), kinetics, computer modeling, Michaelis-Menten, molecular pharmacology

## Abstract

Titrating G protein-coupled receptor (GPCR) signaling to achieve desired physiological outcomes without overdose can be accomplished through partial agonism rather than drug dosage. In this study, we use FRET-based biosensors in combination with ordinary differential equation modelling to identify two key kinetic features that determine agonist efficacy: the concentration-dependent association rate of the G protein to the agonist-bound receptor, and the catalytic rate of G protein activation by the receptor. While both rate constants scale proportionally with reported molecular efficacy metrics, we find that the concentration-dependent association rate is the governing parameter across multiple distinct GPCR-ligand pairings. Our model explains discrepancies in signaling outcomes observed between physiological observations and cellular model systems that rely on receptor overexpression.

G protein-coupled receptor (GPCR) ligands are historically classified from their observed physiological effects. Ligands that stimulate receptor activity are termed “agonists”, whereas those that block the activity of endogenous molecules are termed “antagonists”. Ligands with intermediate physiological effects are referred to as “partial agonists” (1). Such terms are typically defined by effects downstream of the receptor, such as G protein activity, second messenger accumulation, gene transcription, and physiological response. These “top down” attempts to quantify the nature of the ligand-induced response, or “efficacy”, are understood to be system dependent, as they rely on the unique composition/state of each cell or tissue type (2, 3, 4). Consequently, despite promising results in assays commonly used in drug discovery, new pharmacological compounds are often stymied by unpredictable effects when translated into animal or human subjects (5, 6, 7, 8, 9).

In contrast, molecular efficacy attempts to directly measure conformational changes in the receptor and the resulting effects on G protein binding and activation (10, 11, 12). Ideally, this metric is a system-independent parameter that can translate the relative effects of ligands across cell types, model systems, and species. In the quest for this parameter, ligand-stimulated changes in receptor conformational dynamics have now been extensively studied using high-resolution structural biology combined with biophysical approaches (13, 14, 15, 16). However, such measurements of structurally derived efficacy require extensive single-molecule measurements and the examination of time-dependent correlated fluctuations (17). Thus, transforming these “bottom-up” structural insights into downstream responses remains an outstanding challenge.

To address the cellular basis for GPCR signal transduction, substantial effort has gone into modeling the interactions between ligand, receptor, and G protein as an attempt to bridge the structural basis for ligand efficacy with physiological observations (18, 19, 20). The panoply of interaction states populated by ligand, receptor, and G protein that lead to productive GPCR signaling has been captured in the cubic ternary complex model and a number of increasingly detailed iterations thereof (10, 11, 12, 21, 22). While individual on/off rate constants for components of these models have been successfully measured in live cells, these models are limited by the need to quantify operational parameters that define cooperativity in complex formation and the convolution of cellular measurements with protein expression levels (23, 24).

The quest to quantify agonism is further complicated by seemingly counterintuitive behavior observed for certain compounds. Although traditionally classed as antagonists, these compounds possess so-called intrinsic sympathomimetic activity (ISA), whereby in spite of blocking the agonist binding, some positive signaling response is still present (25). By previous definitions, compounds with intermediate activity would be classed as partial agonists. Recently, this argument was brought to the forefront in the context of determining the mechanism of the prescribed cardioprotective agent carvedilol (26, 27, 28). While this discussion has largely focused on the subjective identity of a compound, single-molecule work has also shown that the kinetics of the states induced by some of these compounds exhibiting ISA are indistinguishable from antagonists (17). Therefore, ligand identity inferred from receptor structural dynamics and signaling responses do not necessarily converge to define cellular pharmacology.

In this study, we use a combination of biophysical assays in a cell-free system and live cell measurements to generate a simplified model of cellular efficacy. First, we measure the relative effects of a range of agonists on two distinct interactions that report on signaling complex formation: receptor coupling to a minimal G protein fragment (miniGs) and receptor coupling to a peptide derived from the c-terminal alpha-helical region of the Gα subunit (S peptide). We find a spectrum of responses for ligands and a weak correlation to designated pharmacological roles across multiple class A GPCRs, including many that unexpectedly stimulate GPCR-G protein interactions despite being classified as antagonists. To provide a quantitative framework for these observations, we propose a simplified kinetic model of GPCR-G protein interactions with two key parameters: association rate (k_f_[G]) and catalytic rate (k_cat_). We derive a proxy for k_cat_ from the strength of interaction with the Gα subunit C-terminus and infer k_f_ from both *in vitro* and live cell measurements. Our simplified model attempts to translate receptor-G protein interactions into downstream responses and predicts the association rate as a dominant parameter with implications for the interpretation of second messenger signaling data.

## Results

**Figure 1:**
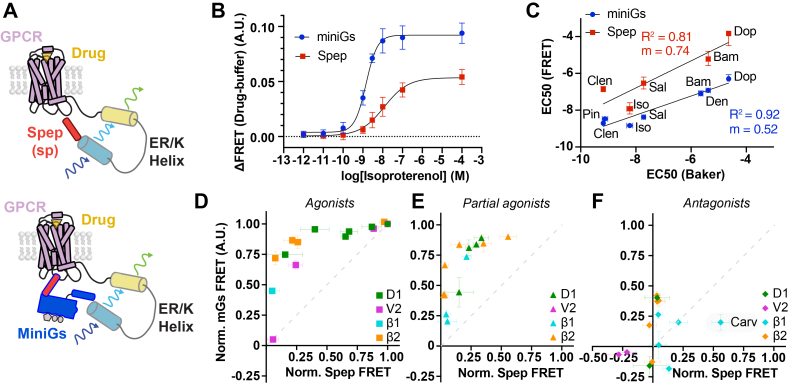
**MiniGs and the S peptide differentiate classes of ligands.***A*, schematic of the design of fusion FRET SPASM sensors used. *B*, dose–response curves for β2AR miniGs and S peptide sensors in response to isoproterenol stimulation. Points are the average of N ≥ 3 points consisting of 3 to 4 technical repeats each, error bars represent standard deviation. Plotted lines indicate fit of points to a concentration-response relationship using nonlinear regression (Experimental procedures). *C*, comparison of previously published EC_50_ data (34) with empirically determined data collected with our FRET sensors. Plotted lines indicate linear fits with the depicted R^2^ values. *D–F*, Normalized FRET responses of s peptide (x axis) and miniGs (y axis) of the indicated ligand classes as described by IUPHAR (see Experimental procedures) for each of the indicated receptors. Dashed lines (*grey*) have a slope = 1, indicating a theoretical correlation of 1. Points are the average of N ≥ 3 points consisting of 3 to 4 technical repeats each, error bars represent standard deviation.

We have previously used a cell free assay to demonstrate that receptor interactions with a peptide derived from the c-terminus of the GαS subunit (S pep) linearly correlate with the molecular efficacy triggered by ligand binding (29). To address the cellular mechanisms of ligand efficacy, we modified this assay to probe the interaction between receptor and miniGs protein, which mimics the GPCR-Gα subunit interface and has been used extensively to derive structural insights into receptor-G protein interactions (30, 31, 32). Briefly, the receptor and S peptide/miniG protein are fused with an ER/K linker flanked by a FRET pair (mCer/mCit) that reports on the strength of ligand stimulated GPCR-S peptide/miniG interaction (Fig. 1*A*). These GPCR FRET sensors are transiently expressed in Expi293F cells and subsequently vesiculated into Giant Plasma Membrane Vesicles (GPMVs) that provide a stable, cell-free system to profile ligand effects (29, 33). Concentration response curves for β2 adrenergic receptor (β2AR) demonstrate a higher FRET response (ΔFRET E_max_), with greater sensitivity to ligand (EC_50_) for miniGs compared to S peptide (Fig. 1*B*). The increased sensitivity (EC_50_) was found to be reproducible across a range of full and partial agonists for β2AR and linearly correlates with previous downstream cAMP measurements (Fig. 1*C*) (34).

We found that across a range of varying efficacy ligands a higher ΔFRET E_max_ for miniGs compared to S peptide is elicited in sensors for four Gs coupled receptors (β2AR, β1AR, D1R, and V2R) (Figs. 1, *D–F* and S1). Relative to a reference full agonist, ligands designated as agonists (Fig. 1*D*) or partial agonists (Fig. 1*E*) show stronger interactions with miniGs compared to S peptide. Antagonists, in general, have no detectable responses for S peptide but a range of modest (normalized ΔFRET< 0.5) responses for miniGs (Fig. 1*F*). A notable exception is carvedilol, which induces a partial agonist-like response (normalized ΔFRET = 0.56 ± 0.07) for β1AR-S-peptide. This behavior is consistent with previous reports of signaling activity induced by carvedilol despite it being classified as an antagonist for this receptor (26, 27, 28).

The higher FRET response for miniGs compared to S peptide is consistent with its larger interface with the receptor (30). To address the relative contribution of S peptide to the miniGs FRET response, the β2AR-S peptide interaction was destabilized through mutations that disrupt intermediate and fully coupled states (35). Mutations that disrupt the β2AR-S peptide interaction (Fig. S2, *A* and *B*) have a marginal effect on β2AR-miniGs sensor responses (Fig. S2*C*). Further, decreasing the effective concentration of the intramolecular interaction with longer ER/K linkers (20 and 30 nm) diminishes FRET responses for S peptide but not miniGs sensors (Fig. S2, *D–G*). Thus, our data suggest that miniGs not only has a stronger interaction than the S peptide, but one that is partially independent of the receptor-S peptide interface.

**Figure 2:**
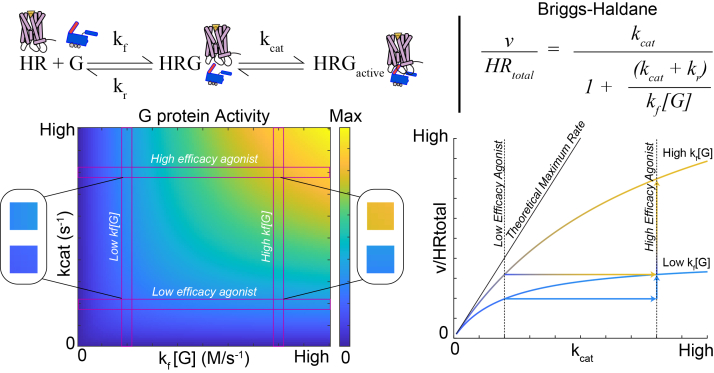
**A simplified kinetic model of receptor/G protein interactions.***Top*, *left*: a simplified kinetic model of receptor (R)/G protein (G) interactions in the presence of saturating ligand (H). The forward and backward rates of association are described by k_f_ and k_r_, respectively. The catalytic rate of activation of the G protein is described by k_cat_. *Top*, *right*: a rearrangement of the Briggs-Haldane equation describing the velocity normalized to the total amount of hormone bound receptor (v/HR_total_) of the reaction depicted to the left. *Bottom*, *left*: a heat map of G protein activity as it relates to the catalytic rate (k_cat_) and the product of the forward rate of association of the G protein with the receptor and the concentration of G protein (k_f_[G]). Warmer colors indicate higher total G protein activity (*i.e.*, cAMP response). *Red boxes* indicate specific regimes of the indicated parameters corresponding to a high or low efficacy agonist and high or low G protein binding and availability. The insets are expanded versions of the highlighted regions at the intersections of the *red boxes*. *Bottom*, *right*: the relationship between the velocity of the reaction normalized to the total amount of hormone bound receptor (v/HR_total_) and k_cat_ at two different rates of association for the G protein. Vertical dashed lines represent low and high efficacy agonists.

To synthesize our findings with these G protein mimetics in the context of receptor-G protein activation, we generated a simplified Briggs-Haldane model of the GPCR-G protein interaction. Briggs and Haldane (36) outlined a generalized form of the kinetic system characterized by Henri, Michaelis, and Menten (37). Whereas Michaelis-Menten kinetics describes a system where the catalytic rate of the enzyme is rate-limiting, Briggs-Haldane makes no such distinction. In our model, hormone-bound receptor (HR) associates with the G protein (G), with corresponding forward and reverse rates (k_f_ and k_r_) governing the formation of a ternary complex (HRG). Association is followed by catalytic activation through interaction with the G peptide (k_cat_) (Fig. 2, *top left*) (38, 39). While the miniGs protein cannot undergo nucleotide exchange, it is assumed to be able to transition to the fully coupled state necessary for G protein activation (30). Our model assumes a regime of proportionality between active G protein and second messenger signaling. This assumption is consistent with the widespread use of receptor and G protein conformational sensors as a proxy for cellular activity of ligands (40, 41). The transition to this state is governed by the rate constant k_cat_ to yield the active complex (HRG_active_). Unlike the cubic ternary complex model, our simplified model does not explicitly consider every possible state of receptor and G protein. Nonetheless, it attempts to capture rate-limiting transitions that are likely to occur for hormone-bound receptors that are biased towards activation of the G protein. This kinetic model is dominated by two factors: the concentration-dependent association rate (k_f_[G]), and the catalytic rate (k_cat_) (Fig. 2, *top right*). Examining the parameter space of resulting G protein activity reveals that agonists with low molecular efficacy (low k_cat_) and systems that are poor at recruiting G protein (low k_f_[G]) have largely similar predicted activity (Fig. 2, *bottom left*). Further, increasing either parameter, while the other remains low, is unable to stimulate significantly higher predicted activity. Hence, our model predicts that agonists with high catalytic rates are unable to trigger significant G protein activity and downstream signaling (*v/HR-total*) unless they also facilitate G protein association (k_f_) and/or operate in an environment with high G protein availability [G]. Together, this model predicts that the difference in activity between high and low efficacy agonists is governed by both the ability of the hormone-receptor complex to recruit G protein and the catalytic rate of the agonist-induced conformation of the receptor/G protein complex (Fig. 2, *bottom right*), as has been shown previously for the calcitonin receptor (42).

**Figure 3:**
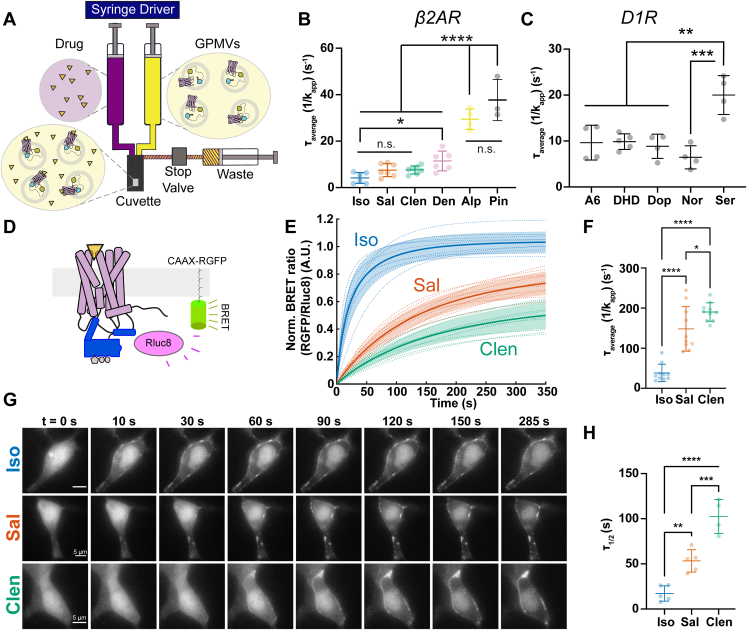
**Determining the relative rate of G protein association.***A*, schematic of the stopped flow apparatus. Briefly, drug and GPMVs containing receptor SPASM FRET sensors are injected by separate syringes and mixed in the cuvette, where FRET is monitored over time. *B* and *C*, calculated T_average_ response times for stopped flow traces, corresponding to the rate of association (1/k_f_) for β2AR (*B*) and D1R (*C*). *Asterisks* represent significant difference as determined by ANOVA and Tukey’s multiple comparisons test. Plotted points are the average of 3 to 4 technical repeats, lines are average and error bars standard deviation. N ≥ 3. *D*, schematic of the bystander BRET assay. Stimulated receptors recruit miniG proteins tagged with Rluc8 to the membrane, which is labeled with CAAX-tagged RGFP. The induced proximity of the Rluc8 allows for quantum energy transfer to the fluorophore, measured as BRET over time. *E*, fraction of maximal isoproterenol BRET response for the indicated ligands measured over time. Solid lines represent the average of the individual repeats depicted as dotted lines. The shaded regions represent the standard deviation of the data. *F*, mean (lines) and standard deviation (error bars) of the T_average_ data calculated from double exponential fits of the traces represented in *panelB*. *Asterisks* represent significant difference as determined by ANOVA and Tukey’s *post hoc* test. Plotted points are the average of 3 to 4 technical repeats, lines are average and error bars standard deviation. N ≥ 10. *G*, representative timelapse images of HEK cells expressing fluorescent miniGs and B2AR (FLAG-mNeonGreen-β2AR, not depicted) stimulated with the indicated ligands. *H*, the calculated t_½_ recruitment times of fluorescently labeled miniGs to the membrane of HEK cells.

To understand the influence k_cat_ and k_f_ have on receptor signaling, we needed empirical measurements of their relative contributions to G protein activation to constrain the variables in our model. Our previous work has demonstrated that receptor-G peptide interactions correlate with GTPase activity at saturating ligand and G protein concentrations (17, 29). Therefore, ligand-induced FRET response of β2AR-S peptide sensors correlate with the catalytic rate of G protein activation (k_cat_). To characterize the forward rate of receptor-G protein association (k_f_), we used β2AR-miniGs sensors to mimic the rate of ligand-stimulated ternary complex formation. Neither of these measurements are a direct substitute for measurements of the theoretical parameters of k_f_ or k_cat_, which in turn are influenced by the receptor structural dynamics. Instead, we use them as surrogates for these rates, given the observed proportionality between our empirical measurements and our theoretical model parameters. Utilizing the *in vitro* GPMV platform, a stopped flow apparatus was used to monitor FRET sensor kinetics after the addition of saturating amounts of drug (Fig. 3*A*). For β2AR, the partial agonists/antagonists denopamine, pindolol, and alprenolol all recruited miniGs significantly slower than the full agonist isoproterenol (Figs. 3*B* and S3*A*). When analyzed separately, we also detected significant differences between isoproterenol, and the partial agonists salbutamol and clenbuterol (Fig. S3*B*; Iso: 4 ± 2; Sal: 8 ± 3; Clen: 8 ± 2; Ratio ∼ 1:2:2). These data were supported by similar observations for D1R, where sensors showed slower responses to the partial agonist serotonin as compared to full agonists (Fig. 3*C*). To exclude potentially confounding effects from the ER/K linker fusions in the sensors used for these experiments, we monitored recruitment of untethered miniGs to the membrane in live cells through bystander BRET (43). Briefly, luciferase-tagged miniGs protein was detected in proximity to the membrane through BRET with a CAAX-labeled rGFP (Fig. 3*D*). Differential recruitment was observed in order of the drugs’ efficacy, with isoproterenol recruiting significantly faster than the partial agonists salbutamol and clenbuterol (Fig. 3, *E* and *F*; Iso: 38 ± 22; Sal: 148 ± 55; Clen: 190 ± 23; Ratio: ∼1:4:5). As another orthogonal approach, miniGs recruitment to the plasma membrane was visually recorded using fluorescent microscopy. Consistent with our *in vitro* GPMV assay and bystander BRET measurements, isoproterenol translocation to the membrane was significantly faster than salbutamol and both were faster than clenbuterol (Fig. 3, *G* and *H*; Iso: 17 ± 9; Sal: 53 ± 12; Clen: 103 ± 19; Ratio: ∼1:3:6). Together, our results support a model in which agonist-stimulated G protein recruitment (k_f_) works in tandem with the catalytic rate (k_cat_) to determine agonist efficacy (Fig. 2, *bottom right*).

**Figure 4:**
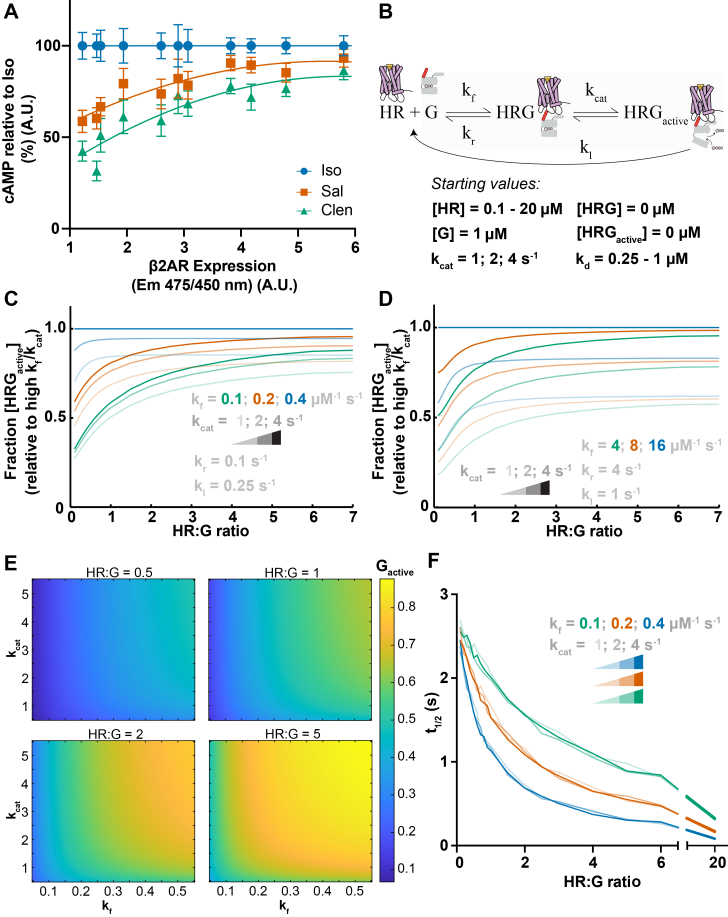
**Expression level dependence of signaling responses.***A*, cAMP response for Iso, Sal, and Clen for increasing expression levels of β2AR. Average response of four technical replicates is indicated by the markers; standard deviation by the error bars; data is collected from three biological replicates; connecting lines are best fits to a nonlinear regression (See Experimental procedures). *B*, modification of simplified kinetic model used to program system of ordinary differential equations. The term kl has been added to account for the rate of G protein recycling through GTP metabolism. Starting rate values and concentrations used in the kinetic model are listed below. *C* and *D*, active G protein generated as a function of receptor expression level for parameter spaces of rate constants dominated by either k_f_ (*C*) or k_cat_ (*D*) depicted as fraction of the maximum modeled rate. Similarly colored lines share kf values. Thick lines show example parameters that co-vary (*e.g.*, a low k_f_ with a low k_cat_, a middle kf and kcat, and a high k_f_ and k_cat_). *E*, a heat map of G protein activity as it relates to the catalytic rate (k_cat_) and the forward rate of association of the G protein with the receptor (k_f_) for different expression levels of receptor. Warmer colors indicate higher total G protein activity (*i.e.*, cAMP response). *F*, the time to half maximal accumulation of bound G protein species for the k_f_ dominated parameter space depicted in *C*. Similarly colored lines share k_f_ values, while opacity depicts the modeled k_cat_ values.

Our simplified model (Fig. 2) predicts that G protein activity depends on the concentration of available G protein, in addition to k_f_ and k_cat_. While it is generally assumed that there is an excess of G protein relative to receptor, cell culture studies often rely on over-expression of the receptor to achieve a measurable downstream signaling response (23, 24, 34). However, receptor overexpression can fail to differentiate agonists based on their expected efficacies, with partial agonists stimulating signaling responses that equate to reported full agonists (34, 44, 45). Our data demonstrate that differences in cAMP levels stimulated by partial and full agonists diminished with increasing receptor expression (Fig. 4*A*). We hypothesized that this saturation phenomenon may be due to depletion of local G protein. To capture this effect in our model, we incorporated the recycling of active G protein to the inactive state due to GTP hydrolysis, which in turn impacts G protein availability for receptor binding (Fig. 4*B*).

In contrast to the simplified model (Fig. 2), which explored the sensitivity of response to a conceptual parameter space, here we sought to incorporate empirically derived parameters for different agonists (k_cat_, K_d_) to reconstruct experimental measurements (Fig. 4*A*). Catalytic rates for G protein activation were estimated from FRET measurements of full agonist-dependent G protein activation through heterotrimer dissociation (∼1–3 s^−1^) (23, 46, 47, 48). The catalytic rates of GTP hydrolysis by G proteins, stimulated by β2AR for the partial agonists salbutamol (Sal) and clenbuterol (Clen) respectively, have been reported as twofold and fourfold lower than the full agonist isoproterenol (Iso) (17, 29). Hence, we combined these reports to model k_cat_ over a range of 1 to 4 s^−1^. Next, the transient nature of G protein-receptor interactions was modeled as having ∼ μm K_d_ (17). Our experiments with β2-miniGs using full and partial agonists had indistinguishable E_max_ levels of FRET for Iso, Sal, and Clen, suggesting these states of agonist bound receptor each have a similar K_d_ for the G protein (Figs. S1 and S2*E*). Likewise, the effective concentrations of receptor and G protein confined to the membrane were assumed to be in the micromolar range. Together, the effects of these parameters (k_cat_, K_d_) on active G protein levels (HRG_active_) across a range of receptor expression levels was determined through a series of ordinary differential equations (ODEs; see Experimental procedure). Our ODE solutions demonstrate the temporal evolution of different receptor:G protein association states (Fig. S4). High receptor concentrations, combined with fast association rates (k_f_) result in a notable peak in HRG, consistent with a lag in G protein activation (HRG_active_) predicted by more complex models (18).

Our simplified model suggests that receptor responses are equally sensitive to k_f_ and k_cat_ (Fig. 2). Hence, we examined two parameter spaces, one dominated by k_f_ (Fig. 4*C*) and the other by k_cat_ (Fig. 4*D*). To achieve a k_cat_ dominated system, we substantially increased k_f_ with an equivalent increase in k_r_ to maintain the experimentally observed K_d_. Of note, the hydrolysis rate, k_l_ was also increased (0.25 s^−1^ to 1 s^−1^) to avoid system saturation (HRG_active_ = [G]) at low receptor expression levels (Fig. S5). In both cases, we model the formation of HRG_active_ in response to three different ligands with three independent association and catalytic rates based on their covariance observed for the model full and partial agonists Iso, Sal, and Clen (see Fig. 3). Given the resiliency of the trends to changes in model parameters (Figs. S6, S7) and the nature of our measurements as proxies for real constants derived from changes in receptor conformation, we chose not to follow one specific assay. Instead, we use a ratio of 4:2:1 for k_f_ to capture the overall relative behavior of the system. The dissociation constant (K_d_) does not influence the trends in active G protein levels (HRG_active_) with varying receptor expression (Figs. S8, S9). Importantly, only for a k_f_ dominated system (Fig. 4*C*) do our estimated model parameters result in plots that capture experimentally measured data (Fig. 4*A*). These findings suggest k_f_, and not k_cat_, determines downstream signaling response.

Examining the full parameter space (Fig. 4*E*), further emphasizes the dominance of k_f_ rather than k_cat_ in tuning the levels of active G protein (HRG_active_). At low expression level ratios (HR:G < 2), we observe a gradient of G protein activation along the k_f_ axis that reflects the predicted cellular response to different efficacy agonists while showing minimal differences with respect to k_cat_. In contrast, at high ratios of receptor expression (HR:G > 5), G protein activation is quickly saturated with increasing k_f_ and k_cat_. To further understand the kinetics of G protein activation, we computed the time to half-maximal association of the receptor:G protein complex (t_1/2_; Figs. 4*F* and S10). As expected, t_1/2_ is dependent on the forward association rate (k_f_) and decreases with increasing receptor expression (Fig. 4*F*). Interestingly, high receptor levels result in t_1/2_ values (∼50 ms) comparable with previous reports from receptor overexpression studies (23). Further, the sensitivity of t_1/2_ to k_f_ rather than k_cat_ is also observed for the k_cat_-dominated parameter space (Fig. S10).

## Discussion

Here, we have demonstrated that for a hormone-bound receptor, a simple model derived from classical Briggs-Haldane kinetics sufficiently captures two key variables that describe ligand efficacy: the catalytic rate of G protein activation by the receptor (k_cat_) and the concentration-dependent association rate of the G protein with the receptor (k_f_[G]). Further, we found the system to be rate-limited by both the association rate and the concentration of available G protein. In this regime, our kinetic model was able to mimic the behavior of distinct agonists with graded efficacy across a range of receptor expression levels. We propose that our simplified model of GPCR-G protein interactions, paired with cell-free sensor measurements, provides a robust, quantitative framework to report ligand efficacy, with the potential for precision targeting of signaling outcomes in drug development.

The physiological effects of GPCR ligands have traditionally been quantified using the operational model of agonism (49). This model combines ligand affinity for the receptor with its concentration-dependent responses, such as second messenger or cell/organ specific measures, to derive an efficacy parameter that enables quantitative comparison of different ligands. However, as the parameters used to define efficacy are dependent upon the specific biological system in which they are measured, translating efficacy across cell culture, primary cell, and model systems has limited the predictive potential of the operational model (2). To address this limitation, a system-independent measure of efficacy, molecular efficacy, has been derived from the structural basis of ternary complex formation and G protein activation (10, 11, 12). However, the focus of molecular efficacy on G protein activation does not incorporate the kinetics of G protein association and turnover in the cellular microenvironment. In contrast, the kinetic model presented here combines both the system-independence of molecular efficacy (k_cat_) and the system-dependent measurements of receptor-G protein association (k_f_[G]) as two separate parameters to describe the effects of ligand-induced signaling.

Ligand efficacy is apparent at different levels of the GPCR signal transduction cascade. At the receptor level, single molecule observations of the cytosolic ends of receptor transmembrane domains reveal agonist-dependent differences in amplitude and/or rates of TM4-TM6 motions that correlate with G protein activation rates (17). Given that this phenomenon can be observed regardless of the presence of G protein, ligand efficacy can be measured at the level of receptor conformation. However, measuring efficacy through the lens of receptor structure requires extensive single-molecule measurements, with the dynamic range of data limited by the fast conformational transitions between inactive and active receptor states. In contrast, the original Black-Leff operational model provides an efficacy metric, tau (t), that combined with the ligand affinity (Ka) yields the classic hyperbolic ligand dose response observed in different cell/organ systems (49). A constraint on interpreting tau and its associated exponent (n) is their system dependence, therefore limiting translation across model systems.

In this context, our study bridges receptor conformation and downstream responses captured by the Black-Leff model to demonstrate that relative ligand efficacy can also be inferred from receptor-G protein interactions. The FRET data for miniGs and S-peptide presented here are surrogates for G protein association and activation, as direct measurements of these parameters require purified recombinant receptor and G protein at multiple concentrations. Hence, while our study is not intended to provide a universal framework for quantitatively defining efficacy, it identifies key, rate-limiting parameters using an accessible experimental system. Combined, this approach provides a qualitative, system-independent basis for comparing ligand-stimulated receptor activity.

In exploring the parameter space of our model, we incorporated rate constants that were reflective of our empirically-derived results and were also consistent with previously published experimental measurements in live cells. Specifically, we assume a forward-biased reaction that proceeds to G protein coupling, a hallmark of the ligand-stimulated signaling cascade. Following agonist stimulation, G protein association with the receptor proceeds at a k_f_-dependent rate to form [HRG]. For a forward biased reaction, HRG proceeds to HRG_active_ rather than dissociate to HR and G. This requires that the catalytic rate (k_cat_) be significantly greater than the dissociation rate (k_r_) to facilitate G protein coupling (Fig. 2). Under these conditions (k_r_ << k_cat_), Briggs-Haldane kinetics (Fig. 2, *top right*) dictate that the precise k_r_ value does not influence system behavior. Further, we model an active G protein recycling rate (k_l_) that is sufficiently high to continue to fuel the system. If this rate is restrictively low (k_l_ << k_cat_ or k_r_), the system becomes saturated with G_active_, regardless of the other rates and concentrations involved. However, if the rate is arbitrarily high (k_l_ >> k_cat_, k_r_), the recycling rate limits active G protein accumulation, thereby compromising the dynamic rate of signaling. Under physiological conditions, hydrolysis of GTP by G proteins is primarily stimulated by the activity of GAPs (50). *In vitro*, GAPs have been demonstrated to be very efficient at stimulating GTP hydrolysis activity in G proteins - a rate reflected in the relatively rapid signal attenuation observed in real biological systems (k_l_ ∼ 10 s^−1^) (51). However, these rates are at odds with some measurements of activity taken in live cells which report slower rates of G protein inactivation (k_l_ ∼ 0.1 s^−1^) (23). These differences may be explained by downstream signaling effects of βγ or internalization of the receptor - which has been associated with both attenuation and compartmentalized signaling. Nonetheless, our model incorporates rates of G protein recycling that reflect downstream second messenger signaling (Fig. 4*A*).

Hein *et al.* (23) have measured significantly faster rates of G protein recruitment compared to our empirical results (Fig. 3). Our slower observed rates are likely to be influenced using miniGs, which is not natively localized to the membrane. Gs protein is natively tethered to the plasma membrane by post-translational modifications and thus is presumed to be constrained to two-dimensional diffusion (52). In the context of the SPASM sensors used for stopped flow measurements (Fig. 3, *A–C*), the rates of interaction are markedly faster, reflecting the higher effective concentration that can be enforced by tethering. However, our qualitative observation that partial agonist-bound receptors recruit G proteins at a rate proportional to the agonist’s efficacy was observed to hold across multiple different methodologies (Fig. 3). In addition, our model is also able to account for faster recruitment rates that are similar to those from previous reports in the context of higher levels of receptor expression relative to G protein (Fig. 4*F*).

Previous studies have measured both the catalytic rate (k_cat_)—derived from measuring G protein activity at saturating levels of G protein (17, 23)—and association rates (k_f_)—measured from the concentration-dependent recruitment rates of miniGs protein to membranes (23, 53)—for distinct agonists. These studies propose two distinct mechanisms for agonist efficacy by examining the correlation between agonist parameters (k_cat_ and/or k_f_) and cellular response (G protein activity). Given the observation that k_f_ appears to co-vary with reported measurements of k_cat_ (17, 29) across agonists, their relative impact on cellular response can only be resolved by a model that incorporates both parameters. The model presented here does this while also describing the dependence of downstream receptor signaling (*i.e.* cAMP) on the available concentration of G protein.

In our model, low levels of active receptor, either from low endogenous expression or low ligand occupancy, are governed by agonist-specific association rates between receptor and G protein. A transition to k_cat_-limited G protein activity is possible by enhancing receptor-G protein association through receptor overexpression or compartmentalized signaling in microdomains. Receptor overexpression in cell culture models has been reported to result in similar second messenger accumulation levels for partial and full agonists. However, a k_cat_-dominated system does not provide for G protein activation on physiologically relevant times scales. Specifically, constraining k_f_ values to experimentally measured G protein association rates, the resulting low k_cat_ values imply ∼ 30 s for each G protein activation. Such response times would be too slow to be useful for the rapid responses induced by biological agonists such as adrenaline or dopamine. However, there may exist some GPCRs for which signaling is not rapidly triggered and may exist in a different regime.

Paradoxically, we observe efficient G protein association with the receptor in response to some ligands classified as antagonists based on minimal downstream response (Fig. 1*F*). In this context, we propose that true antagonists bind the receptor more tightly than endogenous ligands, fail to stimulate G protein recruitment, and have no catalytic activity (low k_f_ and k_cat_). Compounds labeled as antagonists that exhibit ISA instead have low catalytic activity, but a detectable ability to recruit G protein. Partial agonism would then be described by the regime in which both k_f_ and k_cat_ increase until k_cat_ is no longer limiting. Hence, the switch from full agonism to partial agonism and antagonism could then be defined by the shift from a k_f_ constrained system to one that is k_cat_ limited.

## Experimental procedures

### Constructs

The general design of the receptor plasmid constructs used in this paper is as follows: a GPCR followed sequentially by mCitrine (YFP), a 10 nm ER/K linker, mCerulean (CFP), and finally the peptide or miniGs protein. Domains were separated by 2 to 3 Gly-Ser-Gly linkers to promote conformational flexibility and free rotation. rGFP-CAAX was constructed based on the design described by Namkung *et al.* (43). All of the above constructs were cloned into pcDNA 5/FRT. Luciferase-containing miniGs constructs were a kind gift from Nigel Bunnett’s lab. Human β1AR, D1R, V2R (1–345), and V1aR were acquired from DNASU. V2R was cloned with a truncated tail (residues 1–345) to prevent constitutive internalization (54). Human β2AR was acquired from OpenBioSystems (ThermoFisher). Mouse CB1 receptor was acquired from Transomic Technologies.

### GPMV preparation

Expi293F cells (a kind gift from the Lefkowitz lab) were routinely cultured in Expi293 medium (ThermoFisher) at 37 °C and 8% CO_2_ with shaking at 125 rpm (19 mm orbit) in volumes of 30 ml in 125 ml glass flasks. Cells were transfected between 3 and 5 million cells/ml in 30 ml cultures with 3 ml of Optimem (ThermoFisher) containing 30 μg of DNA and 160 μl polyethylenimine (PolySciences). Approximately 48 h post transfection, cells were harvested by centrifugation for 5 min at 350*g*, resuspended in 20 ml phosphate buffered saline, pelleted again for 5 min at 350*g*, resuspended in 10 ml GPMV buffer (1 mM HEPES pH 7.4, 150 mM NaCl, 2 mM CaCl_2_), and pelleted again for 5 min at 350*g* before a final resuspension in 40 ml GPMV buffer. Cell suspensions were divided in half into fresh 125 ml glass flasks and incubated with 2 mM N-ethylmaleimide for 2 h at 28 °C with 140 rpm (19 mm orbit) shaking. After incubation, cell suspensions were recombined and harvested by centrifugation at 1000*g* for 2 min, supernatants were set aside and pellets were resuspended in 10 ml phosphate buffered saline. Resuspended pellets were separated by centrifugation at 1000*g* for 2 min and the resulting supernatant was combined with the one from the previous step. A final spin of 1000*g* for 2 min of the combined supernatants was performed to remove any remaining cell debris. The supernatant from this spin was transferred into a new 50 ml conical tube and spun for 40 min at 3220*g*, 4 °C. Pellets from this spin were resuspended in 2 ml of low salt assay buffer (20 mM HEPES pH 7.4, 25 mM KCl, 5 mM MgCl_2_) and spun again for 40 min at 3220*g*, 4 °C. Finally, the pellet was again resuspended in 2 ml of fresh low salt assay buffer to obtain the final preparation of GPMVs.

### Ligand class identification

Ligands were classified based on their description according to the International Union of Basic and Clinical Pharmacology/British Pharmacological Association (IUPHAR/BPS) guide to pharmacology. Where multiple classifications were present for the same ligand/receptor combination, data were reproduced on both axes. For ligand/receptor combinations that were not explicitly described for either β1AR or β2AR, the classification that exists for the missing receptor/ligand pair was used instead. For ligands that were not listed in the guide to pharmacology, classifications by the manufacturer of the compound were used. For ligands that did not have a clearly defined classification from either of these sources (*e.g.*, epinephrine/D1R), a practical determination was made based on physiology and the response of the sensor.

### Fluorescence measurement of GPMVs

The integrity of GPMVs was assessed by fluorescence spectroscopy using an excitation of 430 nm (bandpass 8) and monitoring emission from 450 to 600 nm (bandpass 4) using a Fluoromax-4 fluorometer (Horiba). Samples were diluted such that the maximum CFP emission were approximately 1∗10^6^ counts per second. For endpoint FRET measurements and dose response curves, three to four technical replicates were prepared for each condition on ice in low salt assay buffer and warmed to 25 °C for 5 min prior to their spectra being collected using the above parameters. FRET ratios were calculated as the ratio of YFP fluorescence over CFP fluorescence during CFP excitation.

### Modeling

Mathematical modeling was conducted based on the presented version of the Briggs-Haldane equation. All calculations were carried out in MATLAB (2024b, MATHWORKS). Ordinary differential equations describing our proposed kinetic model were defined as the following:

$$\frac{d\left[HRG\right]}{dt}=k_{f}\left[HR\right]\left[G\right]-\left[HRG\right]\left(k_{cat}+k_{r}\right)$$

$$\frac{d\left[G^{*}\right]}{dt}=k_{cat}\left[HRG\right]-k_{l}\left[G^{*}\right]$$

$$\frac{d\left[HR\right]}{dt}=k_{cat}\left[HRG\right]-k_{f}\left[HR\right]\left[G\right]+k_{r}\left[HRG\right]$$

$$\frac{d\left[G\right]}{dt}=k_{l}\left[G^{*}\right]+k_{r}\left[HRG\right]-k_{f}\left[HR\right]\left[G\right]$$

Starting parameters and assumptions are as defined in the results section of the main text.

### Stopped flow measurements

Fluorescence stopped flow measurements were collected using a Fluoromax-4 fluorometer (Horiba) in conjunction with a KinTek SF-Powermixer. Samples were excited at 430 nm (bandpass 10) and monitored for fluorescence at either 475 nm or 525 nm (bandpass 10) at 100 Hz for cerulean and citrine emission, respectively. Injections were performed after approximately 5 s of baseline data collection and allowed to continue for at least 60 s. Data were analyzed in MATLAB (2024b; Mathworks). Briefly, individual traces were subjected to a 50-point rolling average for automated detection of the injection point. Raw traces were then aligned and trimmed to be of consistent length before averaging traces between technical replicates. Changes in FRET were calculated as the ratio of YFP fluorescence (525 nm emission traces) over CFP fluorescence (475 nm emission traces) with respect to time. FRET traces were subjected to a 25-point rolling average prior to being fit, and a “start” point was determined based on visual inspection for positive changes in FRET signal. Data were fit to a double exponential equation of the form:

$$y=a-be^{\frac{-x}{c}}-{de}^{\frac{-x}{f}}$$

Where a is the maximum FRET response as determined by the average of the final 100 points of the FRET trace and the additional variables constrained as follows: 0 ≤ b ≤ 1; 0 ≤ c ≤ ∞; 0 ≤ d ≤ 1; 0 ≤ f ≤ ∞. Time weighted averages (τ) were calculated as:

$$\tau =\frac{\left(b*c\right)+\left(d*f\right)}{\left(b+d\right)}$$

### Bystander BRET

Bystander BRET experiments were performed in HEK293 Flp-In T-Rex cells (ThermoFisher) cultured in Dulbecco’s modified Eagle medium (DMEM, ThermoFisher) containing 4.5 g/L D-glucose and supplemented with 20 mM HEPES, pH 7.5 (Corning), 10% FBS (ThermoFisher), and 1% Glutamax (ThermoFisher) at 37 °C with 5% CO_2_. For experiments, cells were plated into 6 well dishes at approximately 25% confluency and allowed to recover for 24 h prior to transfection. Cells were transfected using 1 μg of unlabeled β2AR DNA, 0.2 μg of luciferase-tagged miniGs protein DNA, and 1.4 μg of RGFP-CAAX DNA. Transfection reactions were prepared in 450 μl of Optimem I (ThermoFisher) with 30 μl of 1 mg/ml polyethylenimine (PEI, 25,000 kDa; PolySciences) and allowed to rest for 15 min at room temperature prior to addition to the cultured cells. The media containing transfection reagent was removed from the cells after approximately 5 to 6 h and fresh media was replaced. Media was exchanged again 24 h post-transfection. Cells were harvested 48 h post-transfection by removing the media and resuspending the cells in 1 ml of bystander BRET buffer (20 mM HEPES, pH 7.4, 135 mM NaCl, 5 mM KCl, 400 μm MgCl_2_, 1.8 mM CaCl_2_, and 5 mM glucose in water). Resuspended cells were spun at 350*g* for 5 min at room temperature. The supernatant was aspirated and the pellet resuspended again in 1 ml of bystander BRET buffer before being spun again at 350*g* for 3 min at room temperature. The supernatant was aspirated again, and the cells resuspended for a final time in 1 ml of bystander BRET buffer. Cells were counted using a Countess II automated cell counter (ThermoFisher) and diluted to approximately 7.5∗10^6^ cells/ml. 90 μl of diluted cells were added to individual wells of a white, round-bottom 96 well plate so that there were three sets of wells for each condition to be tested. 250 μl of 30 μm drug or buffer was added to a fourth set of wells. The plate was allowed to rest at room temperature for approximately 3 to 5 min while the luminescent substrate was prepared. Prolume purple (NanoLight Technology) was diluted to 5 μm in bystander BRET buffer before 10 μl was added to each well using a repeater pipet (Eppendorf) to minimize time differences. An initial read of fluorescence (470 nm excitation, bandpass 9 nm; 505 nm emission, bandpass 20) was recorded using a Tecan Spark plate reader. Immediately following this reading, the mode was automatically switched to dual color luminescence and read using light filters (“magenta”: ∼450 nm shortpass/610 nm longpass and “green”: ∼505–540 nm) for 3 min. Following this, the plate was ejected from the machine, and a multichannel pipet was used to add and mix 50 μl of drug or buffer into the monitored wells. The plate was read again using the same dual color luminescence protocol for 5 min. One set of technical replicates for each condition was read at a time and three technical replicates were performed over all for each biological repeat. Data were analyzed *via* custom MATLAB code (2024b; Mathworks). Briefly, data were imported, and a buffer of 20 s was added to each technical replicate to account for the dead time between drug addition and resumption of the reading. Data were then individually normalized to the maximum signal of isoproterenol for the corresponding technical replicate and fit to a double exponential equation of the form described above for stopped flow measurements. Time weighted averages (τ) were calculated as described above for stopped flow measurements.

### Live-cell imaging of miniGs recruitment

HEK293T cells were seeded onto 35 mm glass-bottom dishes (MatTek). The following day, cells were transfected with FLAG-mNeonGreen-β2AR (0.7 μg) and miniGs-tagRFP (0.8 μg), using 6 μl 1 mg/ml polyethylenimine (PEI; PolySciences; 1:4 DNA:PEI) in 100 μl of Optimem. Live-cell imaging for miniGs recruitment was performed the following day. Cells were washed and maintained in imaging medium (Hank’s Buffered Saline Solution (HBSS), 0.2% glucose, 100 μm ascorbic acid) throughout the experiment. Cells were treated with 1 μm agonist while capturing movies at 500 ms for 5 min on a Nikon Eclipse Ti inverted epifluorescence microscope equipped with 100x oil immersion objective (1.4 numerical aperture). Data was acquired using Evolve EMCCD camera (Photometrics) or PCO.edge sCMOS camera and Nikon NIS-Elements software.

### Live-cell imaging data analysis

Data analysis was carried out in FIJI (55, 56). Briefly, a freehand line was drawn on a region of membrane in a responsive cell and a square in the nucleus of the same cell. Total fluorescence was monitored over time for both regions of interest. Membrane fluorescence was normalized to nuclear fluorescence for the duration of the movie to account for photobleaching. The initial point was taken as the minimum of normalized membrane fluorescence and the maximum response as the average of the plateau in signal as determined by visual inspection. The t_1/2_ time was calculated as the time it took to reach one half of the difference between the fluorescence minimum and maximum through linear interpolation of the nearest data points surrounding the calculated difference in signal.

### Expression and cAMP

For all cAMP experiments, HEK293 WT cells were from passage numbers 10 to 27. Cells were seeded at 30% confluency in 6-well plates 16 to 20 h pre-transfection. Cells were transfected with 0.2 to 1.5 μg of DNA (to provide a range of receptor expression levels), 3 μl of X-tremeGENE HP DNA transfection reagent (Roche), and 100 μl Optimem. Additionally, an un-transfected (UT) control was prepared in the absence of β2AR DNA. After 24 h, transfected cells were harvested *via* gentle pipetting and centrifuged at 300*g* for 3 min. Media was removed by aspiration, and pellets were resuspended in 1 ml cAMP assay buffer (phosphate-buffered saline (PBS) with 0.5 mM ascorbic acid and 0.2% (w/v) glucose). Cells were washed once by repeating the previous steps. Cell density was measured (Countess II, ThermoFisher) and diluted to 1.5∗10^6^ cells/ml. Expression of transfected constructs was measured *via* fluorescence (Horiba Fluoromax 4). The metric used to assess the expression of the constructs was the ratio between the mCerulean peak emission (430 nm excitation, bandpass 8 nm; 475 nm emission, read from 450 to 600 nm, bandpass 4) over the optical density (430 nm excitation, bandpass 8 nm; 450 nm emission, read as above). Resuspended cells were incubated in an opaque 384-well flat-bottom plate (Greiner Bio-One) with an equal volume of 2X the concentration of isoproterenol. After 10 min of incubation at room temperature, cells were lysed and processed for the cAMP-Glo Assay (Promega) following the manufacturer’s instructions. Luminescence was measured on a Tecan Spark plate reader (500 ms integration, one measurement per well). Each condition was performed on four independent wells, and the experiment was performed 3 times.

### Statistics

Statistical analyses, including dose response curve fits, were performed using Prism (GraphPad Software). The analysis performed for specific experiments is described in the corresponding figure legends. In general, a minimum of three technical replicates was used to generate a biological replicate, and a minimum of three biological replicates was used to generate a data point. Curve fitting for exponential responses (stopped flow, bystander BRET) was performed using the curve fitting toolbox in MATLAB (2024b; Mathworks).

## Data and materials availability

All data, code, and materials used in the analysis are available upon request.

## Supporting information

This article contains supporting information.

## Conflict of interest

The authors declare the following financial interests/personal relationships which may be considered as potential competing interests: Sivaraj Sivaramakrishnan is listed as an inventor on a published patent held by The University of Minnesota on technology used in this study (US Patent number US20210179692A1).
