# Supplementary material for: Kinetic model of partial agonism reveals cellular basis of ligand efficacy
Source: J Biol Chem. 2026 May 21;302(7):113180. doi: 10.1016/j.jbc.2026.113180 (PMC13284481; doi:10.1016/j.jbc.2026.113180)
Supplement: Supplementary Material [file mmc1.docx]

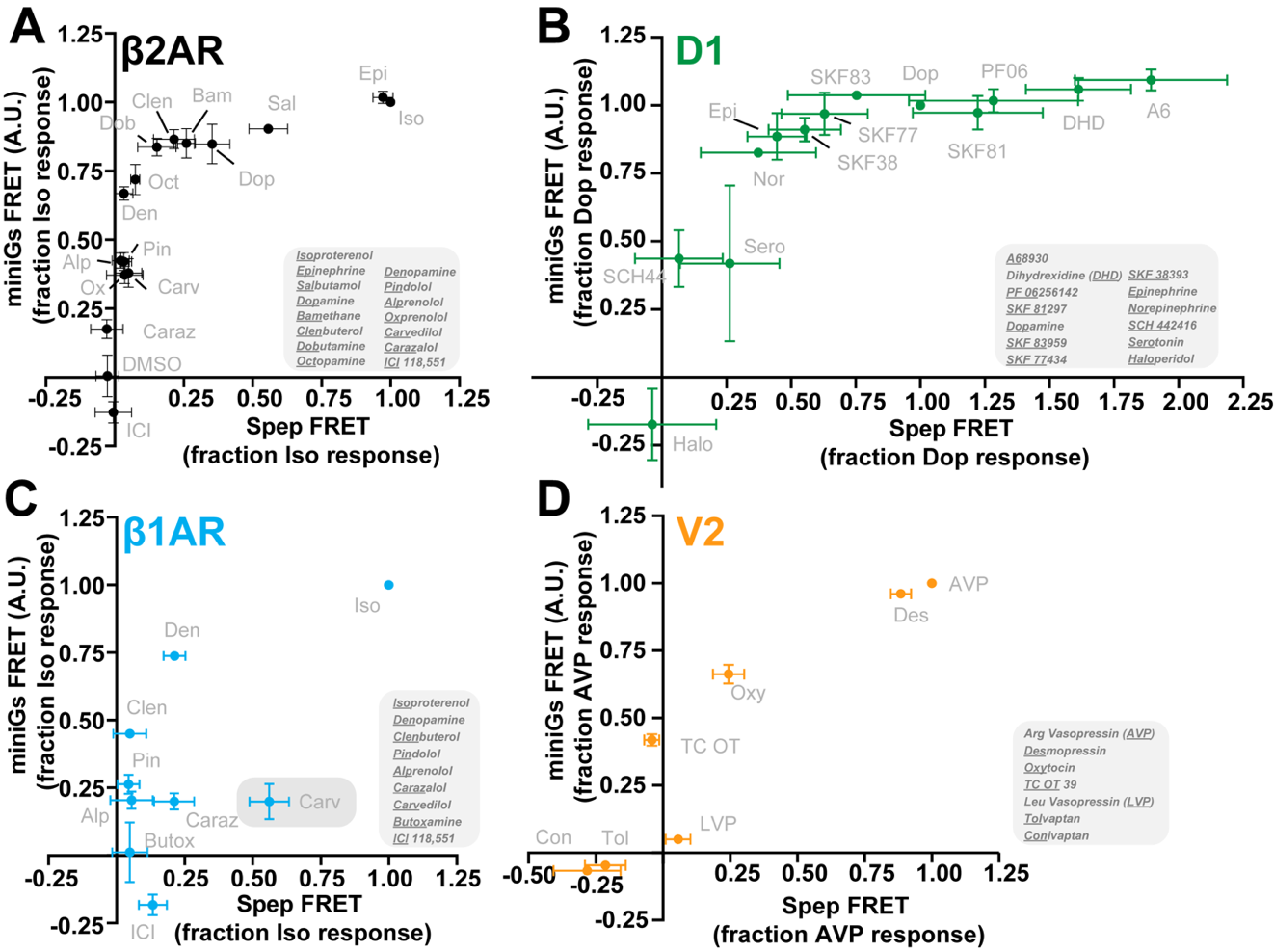


Fig. S1. Similar responses observed across a spectrum of ligands for different receptors. (A) The relationship between ΔFRET for β2ar-S pep and miniGs sensors, represented as the fraction of isoproterenol (Iso) response. Points are the average of at least 3 replicates consisting of 3-4 technical replicates each, error bars are standard deviation. Data reproduced from figure 2 B. (B) The relationship between ΔFRET for D1-S pep and miniGs sensors, represented as the fraction of dopamine (Dop) response. Points are the average of at least 3 replicates consisting of 3-4 technical replicates each, error bars are standard deviation. (C) The relationship between ΔFRET for β1AR-S pep and miniGs sensors, represented as the fraction of isoproterenol (Iso) response. Points are the average of at least 3 replicates consisting of 3-4 technical replicates each, error bars are standard deviation. (D) The relationship between ΔFRET for V2-S pep and miniGs sensors, represented as the fraction of arginine vasopressin (AVP) response. Points are the average of at least 3 replicates consisting of 3-4 technical replicates each, error bars are standard deviation.


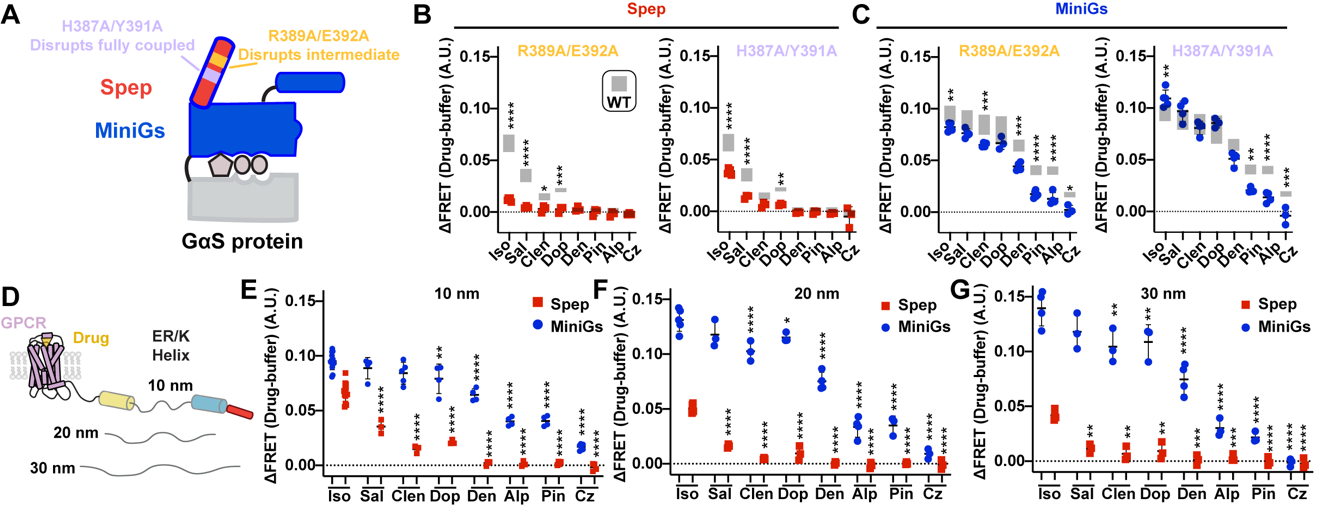
Fig. S2. The interface of miniGs interaction with the receptor extends beyond the orthosteric site of interaction mapped by the peptide.

(A) Schematic of the design and function of SPASM FRET sensors containing mutations in the c-terminal peptide region of either the S pep or miniGs. (B, C) Comparison of the effects of the indicated mutations on S pep (B) and miniGs (C) SPASM FRET sensors. Shaded boxes indicate the standard deviation of data for the equivalent wild-type construct (reproduced from E). Asterisks indicate significant difference from the response of the corresponding wild type construct as determined following a two-way ANOVA and Šídák's multiple comparison test. Plotted points are the average of 3-4 technical repeats, lines are average and error bars standard deviation. N ≥ 3. (D) Schematic of the design of SPASM FRET sensors with different length ER/K linkers. (E-G) The response of constructs with 10, 20, and 30 nm length linkers to a spectrum of full agonist, partial agonist, and antagonist ligands. Asterisks indicate significant difference from the reference agonist isoproterenol as determined following a two-way ANOVA and Šídák's multiple comparison test. Plotted points are the average of 3-4 technical repeats, lines are the average of biological repeats and error bars standard deviation. N ≥ 3


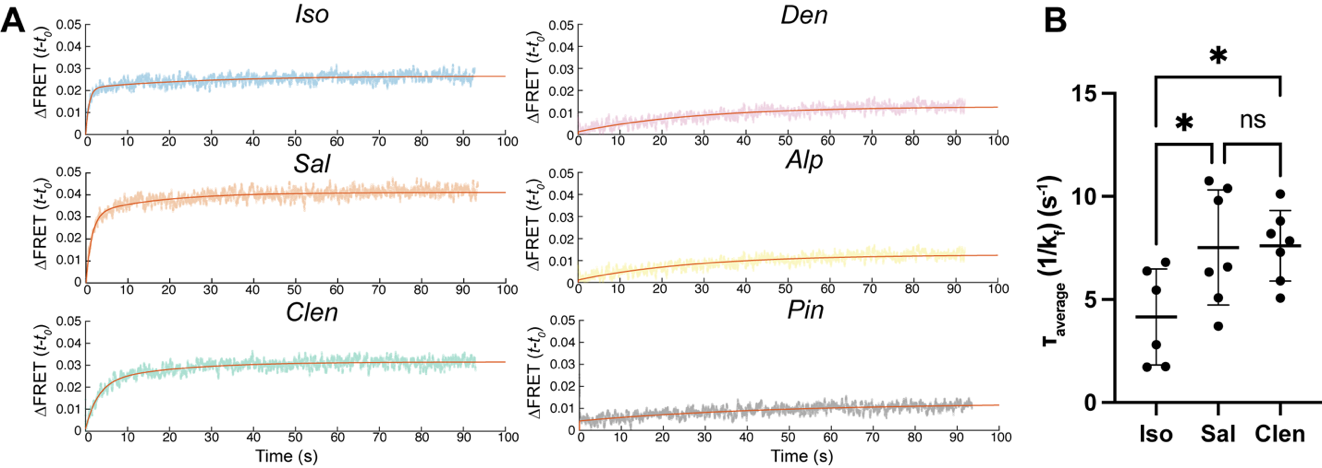
Fig. S3. Example stopped flow trace data and isolated statistical analysis.

(A) Representative traces of ΔFRET data collected with the indicated ligands for β2ar-miniGs SPASM constructs. Plotted data is a 25-point rolling average and the corresponding double exponential fit to the raw data collected at 10 Hz (see methods). (B) Calculated response times (T_average_) for stopped flow traces, corresponding to the rate of association (1/k_f_). Asterisks represent significant difference as determined by ANOVA and Tukey’s multiple comparisons test. Plotted points are the average of 3-4 technical repeats, lines are average and error bars standard deviation. N ≥ 3


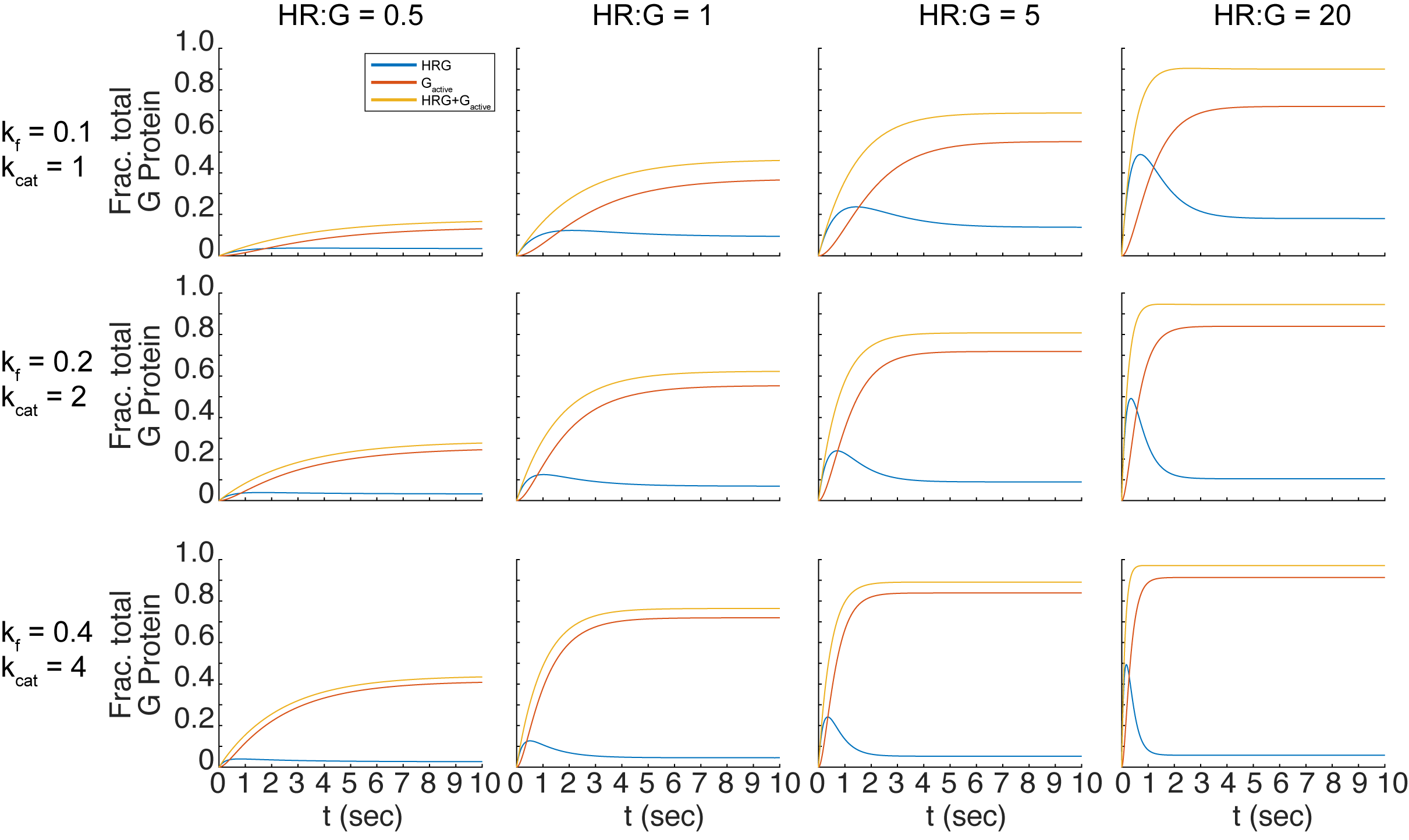


Fig. S4. G protein species generated during modeling of kf dominated parameters spaces at multiple levels of receptor expression. Fraction of total G protein species generated for HRG (blue), G_active_ (red), and both species combined (gold) for parameters in the k_f_ dominated space for k_f_ and k_cat_ values that co-vary.


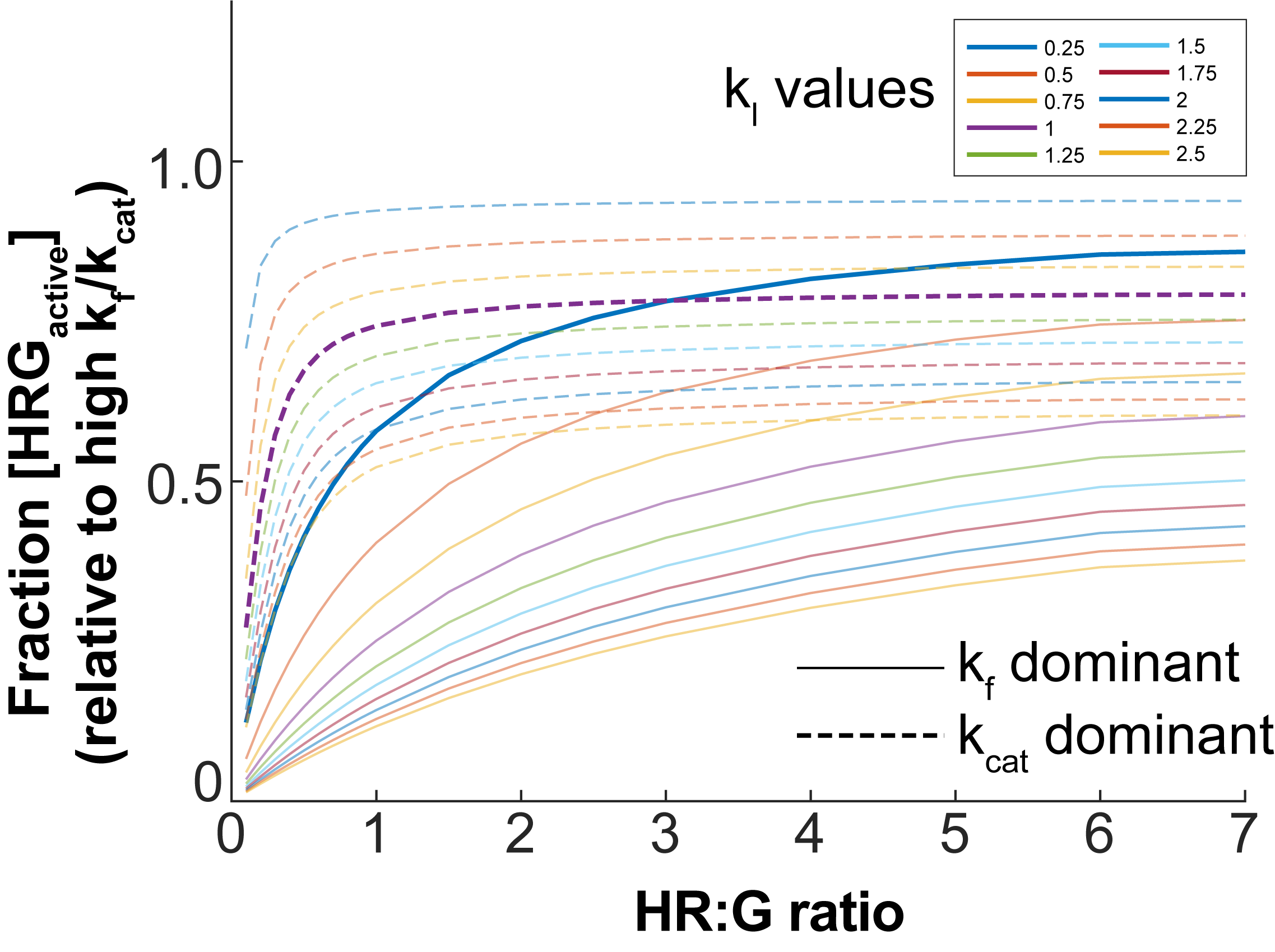


Fig. S5. The influence of kl on g protein activation modeled in kf and kcat dominated parameter spaces. Active G protein species modeled for kf dominated parameter spaces (solid lines) and k_cat_ dominated parameter spaces (dashed lines) at the indicated values of kl (colors) for high k_f_ and k_cat_ values for each space, respectively. Full opacity lines depict the parameter spaces used to model the data presented in Figure 4 C and D.


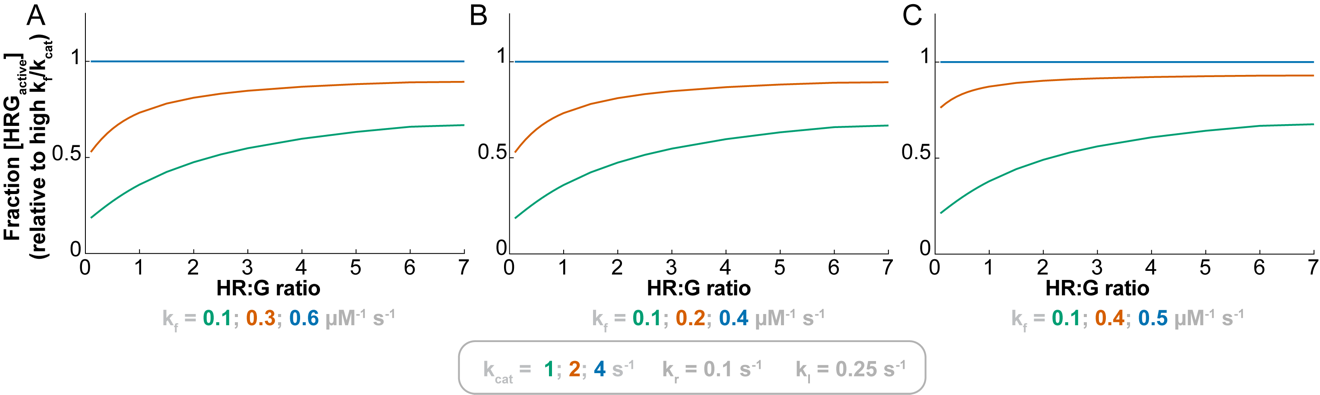


**Fig. S6. Modeled effects of empirically derived values for k_f_.** Active G protein species modeled for the hypothetical ratios of k_f_ measured from microscopy data (**A**) (see Figure 3G, H), bystander BRET data (**C**) (see Figure 3D, E, F) or the representative values used to capture the overall behavior of the system (**B**) (see Figure 4C). Each value of k_f_ is shown for a co-varying value of k_cat_ at the listed values for k_r_ and k_l_ consistent with our assumption of the k_f_-limited space.


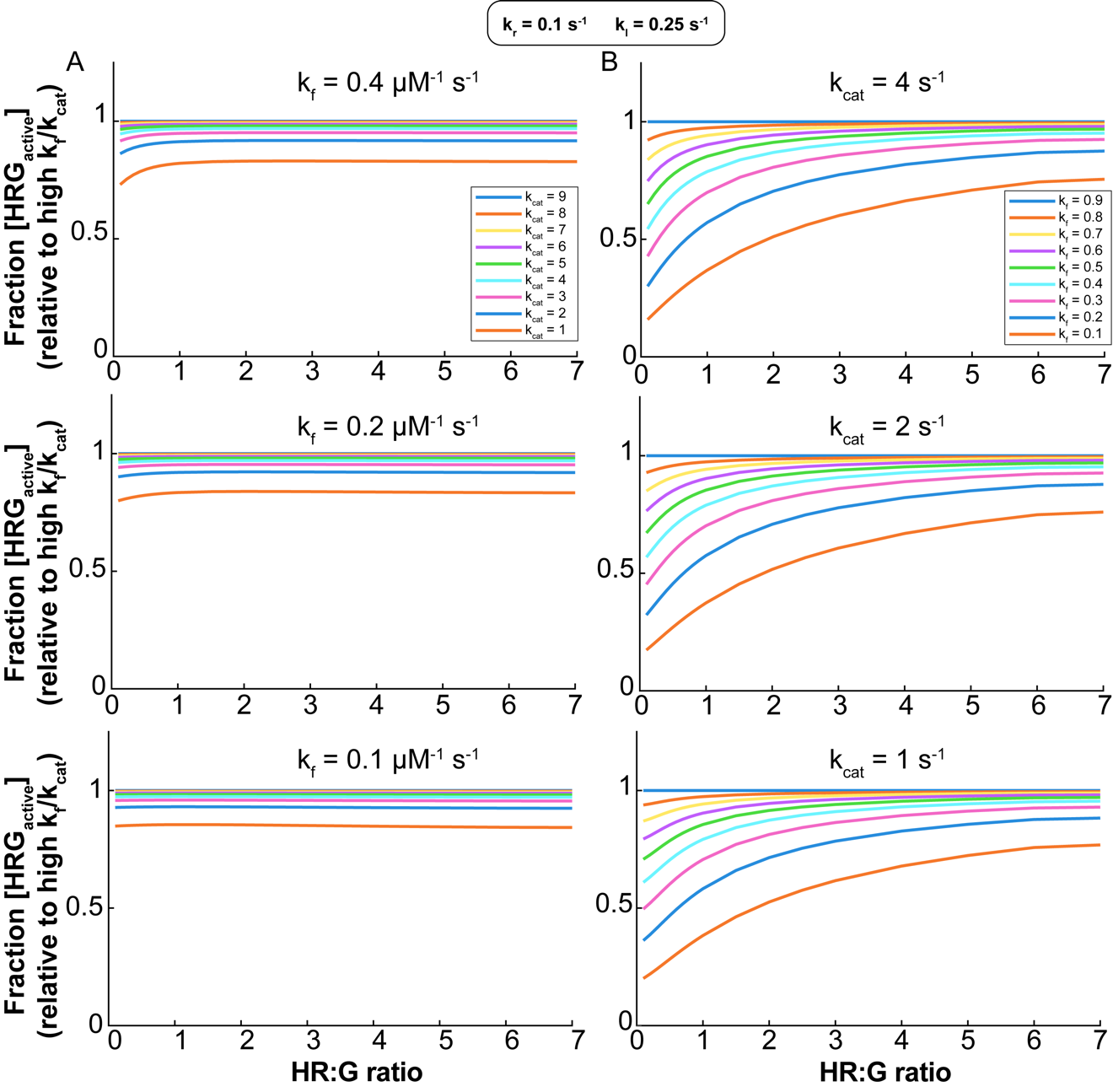


**Fig. S7. Effects of varying k_f_ and k_cat_ on modeled G protein activation.** (**A**) Active G protein species modeled for varying values of k_cat_ at the indicated values of k_f_. (**B**) Active G protein species modeled for varying values of k_f_ at the indicated values of k_cat_. Modeled values of k_r_ and k_l_ are consistent with the k_f_-limited space described in Figure 4C.


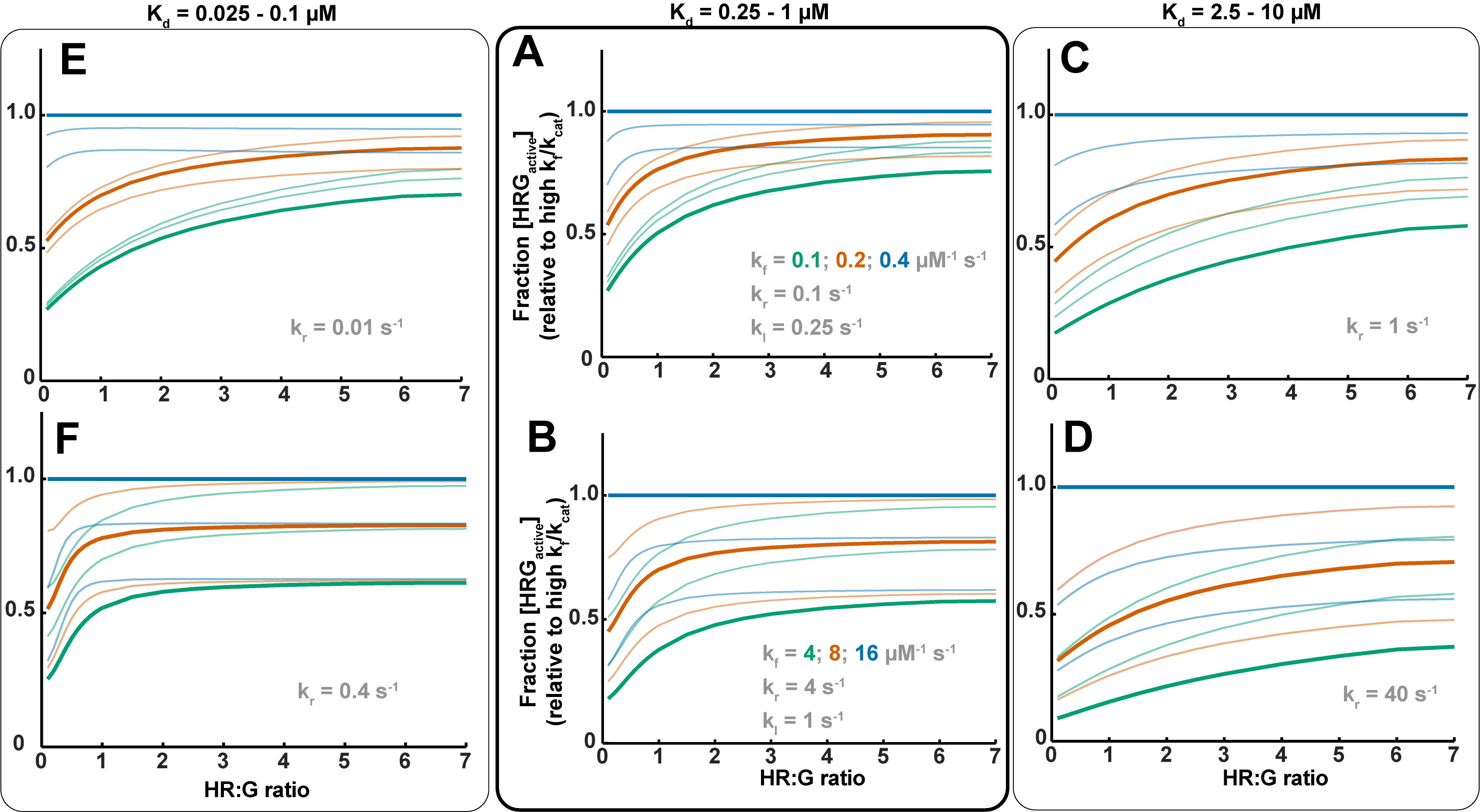


Fig. S8. The influence of k_r_ on G protein activation for a simplified kinetic model of receptor activation. (A, B) The fraction of maximally activated G protein for the given system of parameters for either the k_f_ dominated system (panel A; Fig. 4C) or the k_cat_ dominated system (B; Fig. 4D). (C, D) The same modeled data in panels a and b with a 10-fold higher reverse rate, thereby shifting the K_d_ upward by 10-fold. (E, F) the same modeled data in panels a and b with a 10-fold lower reverse rate, thereby shifting the K_d_ downward by 10-fold.


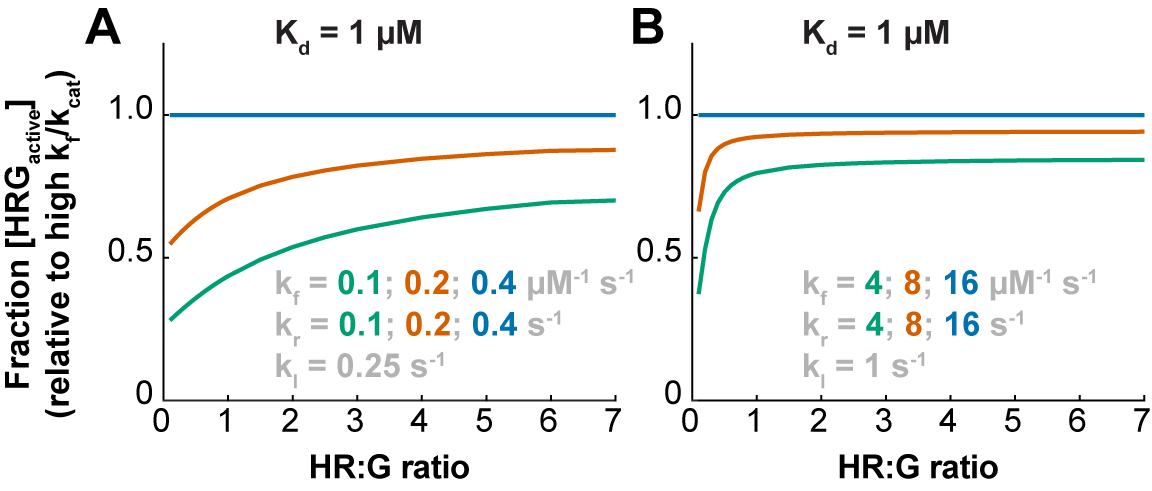


Fig. S9. The effect of constant K_d_ on modeled systems. (A, B) Fraction of maximal active G protein generated when K_d_ is held constant for the modeled parameters in figure 4C and D.


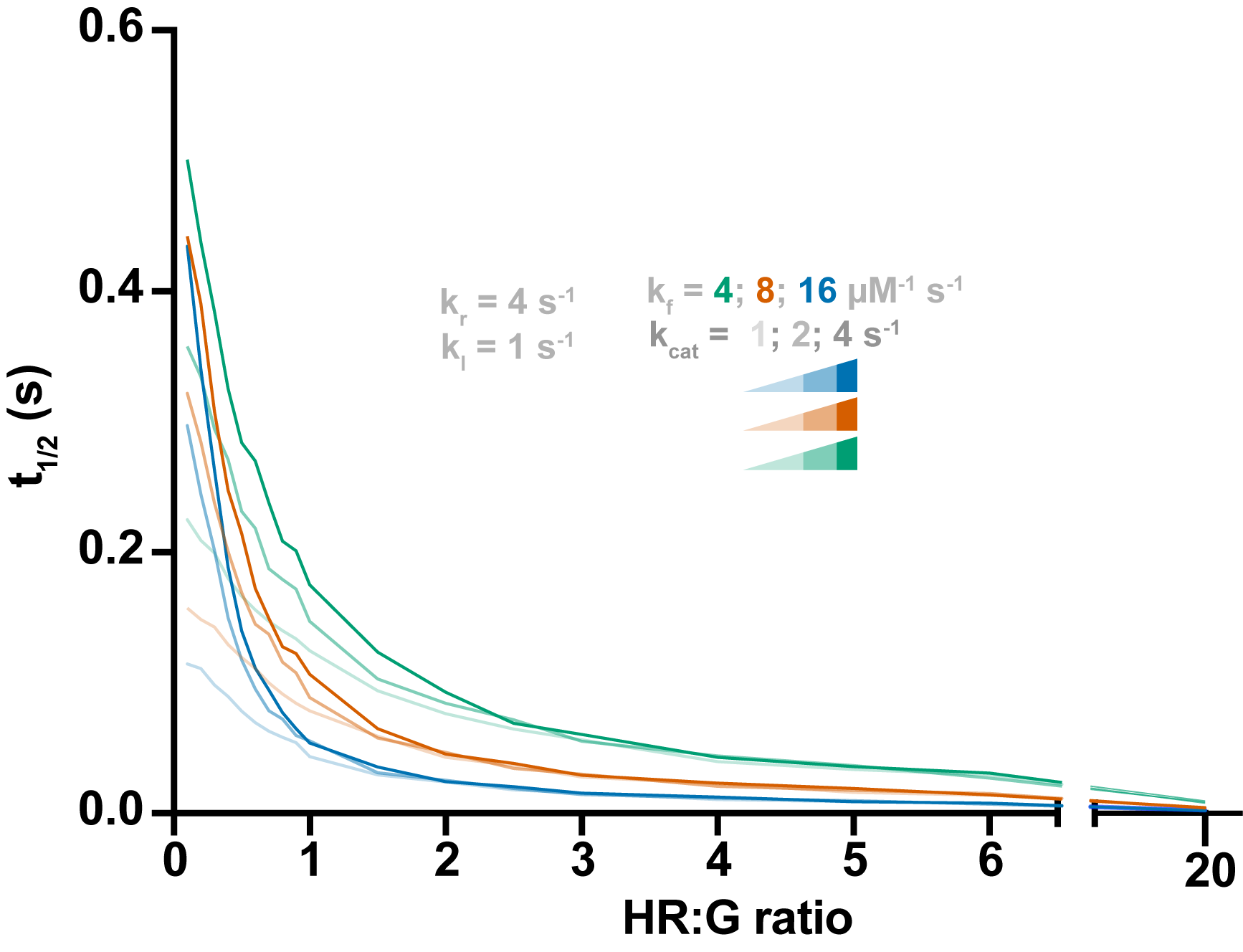


Fig. S10. k_cat_ dominated active g protein accumulation rates continue to segregate on k_f_. The time to half maximal accumulation of bound G protein species for the k_cat_ dominated parameter space (compare to figure 4C). Similarly colored lines share k_f_ values, while opacity depicts the modeled k_cat_ values.
